# Quantitative Nuclear Magnetic Resonance for Small Biological Molecules in Complex Mixtures: Practical Guidelines and Key Considerations for Non-Specialists

**DOI:** 10.3390/molecules30081838

**Published:** 2025-04-19

**Authors:** Eva Drevet Mulard, Véronique Gilard, Stéphane Balayssac, Gilles J. P. Rautureau

**Affiliations:** 1 Institute of Chemistry and Biochemistry (ICBMS), UMR 5246, CNRS, University Lyon, F-69622 Villeurbanne, France; 2 France INSERM Research Unit 1033 LYOS, Lyon 1 University, F-69372 Lyon, France; 3 Laboratoire Softmat, CNRS UMR 5623, Université de Toulouse, F-31062 Toulouse, France

**Keywords:** quantitative NMR, metabolomics, biochemistry, biomolecules, metabolites

## Abstract

Nuclear magnetic resonance (NMR) spectroscopy is a powerful analytical approach that enables both the structural determination and precise quantification of small molecules, such as metabolites. However, achieving precise quantification with NMR involves more than simply comparing integrals derived from NMR peaks to a concentration reference; quantitative NMR (qNMR) is a distinct and specialized application within the field. To obtain absolute quantitative results, spectra must be acquired under strict experimental conditions. Unfortunately, these acquisition parameters can be challenging to implement experimentally and often require trade-offs that compromise high throughput or practicality. In such situations, alternative strategies based on relative quantification and advanced software tools offer valuable solutions. This review aims to provide non-specialists with the key concepts and methodologies required for accurate NMR-based quantification in biomedical research, focusing on practical guidelines and experimental considerations. Unlike prior reviews, it prioritizes accessibility and practical implementation for researchers outside the field, emphasizing key experimental workflows and applications in biological and clinical studies. It clarifies the distinctions between absolute and relative concentration determinations and emphasizes the critical importance of sample preparation, pulse sequence selection, and rigorous control of experimental parameters. Recent technological advancements, such as high-field spectrometers and cryoprobes, have significantly enhanced the sensitivity and accuracy of NMR, enabling the reliable detection of low-concentration metabolites. Quantitative NMR thus offers critical potential in elucidating metabolic processes, supporting drug development, and aiding disease diagnosis.

## 1. Introduction

NMR spectroscopy provides a versatile approach for determining the concentration of molecules both in isolation and within complex mixtures, making it an invaluable tool in numerous fields of biochemistry and biomedical sciences. Over the years, NMR-based quantification applications have indeed been developed and utilized in diverse areas, including enzymology [1], biocatalysis [2], drug discovery [3], metabolomics [4], targeted medical analyses [5], and even in fields such as the food and beverages industry [6], counterfeit detection [7], and environmental and toxicology studies [8]. This review aims to provide practical guidelines and considerations for non-specialists interested in applying quantitative NMR (qNMR) in biomedical and biological research. Unlike previous reviews, which focus on theoretical developments or specific technical innovations in qNMR, this manuscript emphasizes accessibility to researchers outside the field of NMR. It highlights key experimental parameters, practical workflows, and relevant applications in biological and clinical contexts. By bridging the gap between NMR experts and biologists, this review serves as a tutorial-style resource tailored to non-specialists while addressing the current challenges and limitations of the methodology. Prior reviews have extensively discussed advanced NMR methodologies, including Giraudeau’s comprehensive review of qNMR challenges [9]. This manuscript complements such work by focusing on practical implementation and non-specialist accessibility. In particular, it emphasizes the quantification of small hydrophilic molecules, such as metabolites and xenobiotics.

The fundamental strength of NMR lies in the linear relationship between signal area and molecule concentration, enabling the quantification of molecules containing NMR-detectable nuclei. Commonly studied nuclei include ^1^H, ^13^C, ^15^N, ^31^P, and ^19^F. ^1^H NMR has been the most widely used so far, with routine applications to small molecules such as amino acids, sugars, nucleotides, and other metabolites, but also in xenobiotics and organic compounds with a concentration above the micromolar range. Each molecule displays a unique set of NMR peaks, or a “fingerprint”, that reflects its chemical structure and can be used for identification. This fingerprint arises not only from the chemical shifts of the peaks, but also from their fine structure, determining the spin multiplicity and J coupling in the ^1^H NMR spectrum. These features provide additional layers of specificity, enabling the differentiation and identification of analytes. NMR has greatly benefited from the availability of extensive public databases, which catalog spectral fingerprints and facilitate the identification and quantification of biomolecules. These resources, combined with advances in high-throughput NMR metabolomics, now allow for the routine quantification of tens of metabolites (and detection of hundreds) in complex biological samples, like serum or urine [10]. Recent advancements in NMR instrumentation, such as high-field spectrometers and cryoprobes, have significantly enhanced sensitivity and accuracy, enabling the reliable detection of low-concentration metabolites. While high-field spectrometers have significantly advanced NMR sensitivity, recently commercialized benchtop NMR systems would offer a cost-effective alternative, increasing the accessibility of quantitative NMR, particularly when concentration sensitivity is not a limiting factor [11].

While the quantitative capabilities of NMR are well-established, its adoption in broader biological research remains limited due to perceived technical complexity. Determining the concentration of molecules in solution NMR requires stringent experimental conditions, including precise control of acquisition. When these conditions cannot be met, adapted strategies based on relative quantification can be employed. However, there is much confusion between those two approaches, and the perceived complexity of qNMR frequently deters non-specialists from integrating it into their workflows. The development of dedicated software tools, such as the commercial package Chenomx NMR Suite V12 (Chenomx, Edmonton, AB, Canada) or freely available tools, such as Bayesil [12] or Batman [13], has simplified spectral analysis, making NMR increasingly accessible to researchers outside the field of NMR spectroscopy. This article seeks to foster understanding between NMR experts and non-specialists by providing biologists with the key concepts and methodologies necessary for accurate NMR-based quantification in biomedical research. By clarifying the differences between absolute and relative quantification, this review aims to demystify the process and encourage its adoption in biological studies. Additionally, this article highlights the unique advantages of NMR, such as its non-destructive nature and its ability to work on complex mixtures and monitor real-time biochemical reactions, positioning it as a powerful tool for advancing research in metabolomics, drug development, or disease diagnosis.

## 2. Quantification Using NMR, Theoretical Aspects

Quantification using NMR is grounded in the fundamental relationship between the intensity of an NMR signal and the number of nuclei contributing to it. This linear relationship forms the basis of both absolute and relative quantification methods. Unlike qualitative NMR, which primarily focuses on structural elucidation, quantitative NMR (qNMR) demands stringent adherence to theoretical and experimental principles to ensure the accuracy of the quantification results. NMR is considered to deliver highly reliable levels of accuracy under routine acquisition times, accuracy being the combination of precision and trueness [9]. Precision relates to the repeatability (when using the same instrument and operator) or reproducibility (when involving multiple instruments and/or operators) of measurements on the same sample. The precision of NMR is also within the 5% range [14]. Trueness, on the other hand, refers to the closeness of the measured value to the true or reference value. A trueness exceeding 98.5% is typically achievable using NMR with adequate acquisition parameters [15]. Altogether, the trueness and precision of NMR are acclaimed among analytical techniques and often exceed the variability in biological samples, whether from biological or technical origins. Central to these principles are the additivity of NMR spectra, relaxation dynamics, and the necessity of standardized experimental conditions to avoid biases in signal intensities. This section explores the theoretical underpinnings of qNMR, examining the principles that enable quantification and discussing the challenges associated with applying these principles in complex biological and chemical systems. By understanding these theoretical aspects, researchers can better design and execute experiments to achieve quantitative results.

### 2.1. The Additivity Principle and the Possibility of Studying Mixtures of Small Molecules

**Figure 1:**
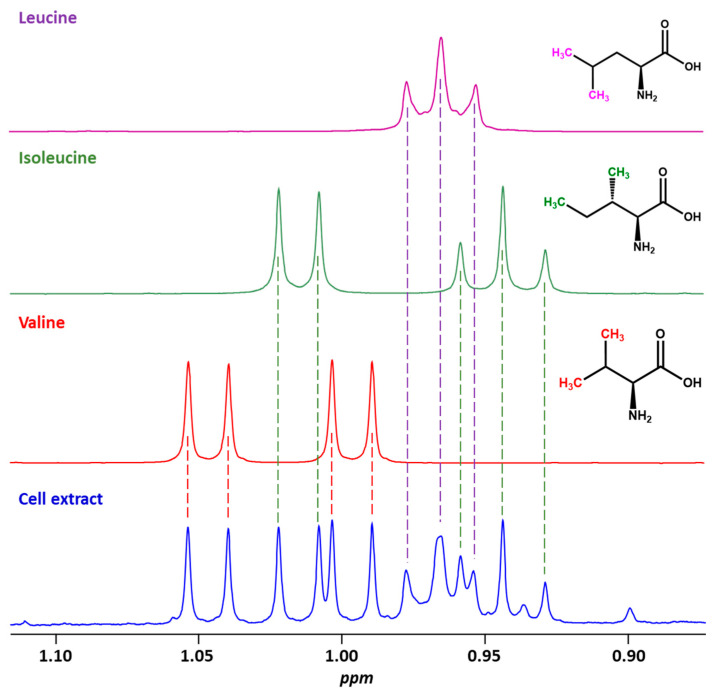
Comparison of ^1^H NMR spectra highlighting methyl signals for leucine, isoleucine, and valine. The methyl groups of each metabolite are readily identifiable in the partial spectrum of the cell extract. Spectra were recorded in D_2_O (pH = 7.4) at 298 K on a Bruker Avance 500 MHz equipped with a TCI cryoprobe.

The additivity principle states that a mixture’s spectrum is the sum of its components’ spectra. This principle, illustrated in Figure 1, is fundamental for analyzing biomolecules within complex mixtures, as it enables the differentiation of each constituent based on its unique spectral fingerprint. By comparing the observed spectrum of a mixture with the spectra of known compounds acquired under identical conditions, biomolecule identification and quantification can be performed with high precision. The assignment process is greatly facilitated by the availability of comprehensive spectral databases, such as the Human Metabolome Database (HMDB) and the Biological Magnetic Resonance Data Bank (BMRB) [16,17]. These resources provide reference spectra and peak assignments for a wide variety of biologically relevant molecules, enabling accurate matching and reducing the ambiguity often encountered in spectral interpretation. Additionally, techniques such as spiking reference compounds into a sample or utilizing 2D NMR to gather insights into peak correlations, coupling patterns, and molecular structures, enhance the reliability of assignments in complex mixtures [18,19].

In practice, the additivity principle underpins most advanced NMR applications, from metabolomics to drug discovery, where complex biological samples like serum, urine, or cell extracts are analyzed. Software tools, such as Chenomx, can deconvolute overlapping peaks and attribute them to specific molecular species. This capability is especially crucial in cases where multiple compounds display overlapping resonances. The additivity principle extends to qNMR, as each compound’s contribution to the overall spectrum is directly proportional to its concentration, provided the experimental conditions are properly controlled.

### 2.2. NMR as a Primary Ratio Quantification Method

NMR spectroscopy is considered a quantitative primary ratio measurement technique, as it allows the direct determination of the ratios of substances in a mixture from the NMR spectrum, without requiring comparison with another compound. The absolute quantities of substances can be established by employing straightforward reference standards [20]. More specifically, under fully relaxed conditions—where the NMR signal is given sufficient time to build up to its maximum or equilibrium state before acquisition (see Section 2.3 for a discussion of the importance and conditions to achieve fully relaxed conditions in practical qNMR experiments)—the integral (or volume in the case of a 2D experiment) of an NMR signal (S) exhibits a direct linear relationship with the number of nuclear spins (N) contributing to that signal [9,21]. This relationship can be mathematically expressed as S = kN, where k is a proportionality constant influenced by various factors, including the sample composition, probe design, acquisition temperature, and other experimental parameters. However, k is identical for all molecules of a mixture within an NMR sample. In practice, the determination of k is achieved experimentally; NMR quantification relies on the use of a concentration reference, which can be either internal (a chemical compound of known concentration added directly to the sample) or external (using an external NMR tube or coaxial inserts to separate the sample and the reference solution) [22]. Once k is determined for a standard molecule through a straightforward calibration process, the quantification of other molecules becomes both accurate and straightforward, provided that the acquisition parameters are optimized for quantitative results and the assignment of spectral peaks or multiplets are established, including the identification of the number of atoms contributing to each peak.

### 2.3. NMR Signal Relaxation and Absolute vs. Relative Quantification

As the relaxation properties of NMR signals differ greatly between molecules, careful consideration of acquisition parameters is essential to ensure that all molecules in the sample contribute fully to the signal intensity. Specifically, all spins contributing to the NMR signal must be fully relaxed before each scan. The T_1_ relaxation time, also known as the spin-lattice relaxation time, is the time constant that describes how quickly nuclear spins return to the thermal equilibrium along the longitudinal (z) axis after the pulse. It is a key parameter that governs the recovery of the signal between scans. If insufficient time is allowed for an NMR signal to relax to its equilibrium state, only a fraction of the total contributing spins will participate in the signal. Consequently, peak integration and the calculated concentration will be underestimated in a metabolite-dependent manner. To achieve quantitative conditions experimentally, adequate pulse sequences and acquisition parameters must be set: for ^1^H spectra measurements, the delay between two scans has to be long enough to ensure that the NMR signal has built up to reach its equilibrium, the total length of the pulses must be as short as possible to minimize signal loss due to relaxation before acquisition [23], and the pulse excitation must be uniform over the spectral width (see the Section 4) [19]. When these conditions are met, all small molecules from the NMR sample will deliver their maximum NMR signal, and any NMR signal can be used as a reference for quantifying others. Under these absolute quantitative conditions, the concentration of compound “*x*” (*C_x_*) is compared with the concentration of standard “*cal*” (*C_cal_*) using the relationship

(1) $$\frac{C_{x}}{C_{cal}}=\frac{S_{x}N_{cal}}{S_{cal}N_{x}}$$

where *S* represents the area of an integral and *N* represents the number of nuclei corresponding to the NMR peak considered [4,9].

If the system is not fully relaxed at the beginning of the experiment, or if the sequence includes long delays that do not allow spins to relax before the acquisition, the absolute quantification conditions are unmet. A metabolite moiety T_1_-dependent signal attenuation will be observed on the NMR spectra and Equation (1) is not applicable (the T_1_ parameter is a metabolite moieties and matrix-dependent parameter that can be measured and that reflects the relaxation properties of the NMR signals of a molecule). Under such non-quantitative conditions, extrapolating the concentration between different compounds from the same sample is thus not possible [24]. It is nevertheless possible to compare integrals of the same signals between samples, thus obtaining relative variations as percentages, with the condition that spectra were acquired under identical conditions and acquisition parameters. The concentration can also be determined under non-quantitative conditions by using a reference for each molecule to be quantified (external reference or spiking experiments in which one or multiple compounds is/are added to an already measured sample). In that situation, the reference allows the direct quantification of the compound, and it is possible to study the same compound across multiple samples, assuming that all experimental parameters remain consistent. Multiple molecule references can be measured simultaneously in the same sample or the same spiking experiment. Their concentration should remain within the same range of concentration and solution conditions as the T_1_ parameter is matrix-dependent. The approach of using references for every molecule to be quantified was chosen by the company Chenomx, which offers dedicated software for metabolite identification and quantification in biological samples [25]. The software relies on a proprietary library of spectra of metabolite standard samples acquired with common (though non-quantitative) parameters, often used in the metabolomics field to reach high-throughput capacity. Under the condition that the NMR spectra are acquired strictly according to Chenomx recommended parameters, the metabolite concentration can be obtained.

In practice, achieving absolute quantification conditions in NMR is challenging and may sometimes be impossible, for example for advanced, long, multi-pulse NMR experiments that require signal selection, water suppression, or multidimensional experiments and high signal-to-noise ratios. However, relative quantifications can always be obtained using specific references or dedicated software. Implementing accurate quantification for such advanced sequences necessitates the use of appropriate calibration methods [19].

While NMR is inherently an additive technique, meaning that signal intensity is proportional to the number of resonant nuclei present, several factors can lead to deviations from this ideal behavior. In particular, differences in T_1_ and T_2_ relaxation times between molecules of varying molecular weights can introduce molecule-dependent signal attenuation, leading to potential bias in quantification. Additionally, non-uniform aggregation and sample matrix effects can alter the effective concentration of molecules in solution, impacting signal linearity. Furthermore, variations in instrumental setup and calibration between different NMR facilities can introduce discrepancies in quantitative results. To mitigate these issues, careful attention to relaxation delays, standardized experimental protocols, and rigorous validation using external or internal references is recommended [26].

## 3. Applications of Quantitative NMR in Biological and Biomedical Research

Quantitative NMR (qNMR) has become an indispensable tool in biomedical research, offering precise and non-invasive quantification of metabolites and other small molecules in complex biological samples. However, it is important to distinguish between two main approaches in qNMR: targeted quantification, which involves the measurement of specific molecule(s) of interest, and untargeted metabolomics, which involves profiling a broad range of metabolites in complex matrices. The latter poses distinct challenges, such as overlapping signals and a wider dynamic range of concentrations, making data analysis more complex and requiring advanced experimental strategies. In clinical metabolomics, qNMR has facilitated the identification of biomarkers for numerous diseases, such as diabetes, cancer, and cardiovascular disorders, providing insights into metabolic pathways and aiding in disease diagnosis and monitoring. For instance, qNMR has been used to quantify metabolite levels in diabetic patients and to detect metabolic shifts in cancer, such as elevated choline and lactate concentrations, which reflect tumor metabolism and hypoxia [4,5,27,28,29]. The technique also plays a critical role in enzymology, enabling the real-time monitoring of enzyme kinetics and reaction progress by quantifying substrate consumption and product formation [1,30]. Such applications are particularly valuable in studying enzyme inhibitors and designing therapeutic agents. Similarly, qNMR has advanced biocatalysis research by quantifying reaction yields and identifying key intermediates in catalytic cycles, providing insights into the efficiency and specificity of enzyme-mediated processes in both industrial and biomedical contexts [2]. In drug discovery and pharmacokinetics, qNMR has proven essential for quantifying drug compounds and their metabolites in plasma or urine, in order to evaluate pharmacokinetic and bioavailability profiles [3]. For example, researchers have applied qNMR to study the metabolic fate of chemotherapeutic agents, enabling the accurate monitoring of drug clearance and identification of low-concentration metabolites [31]. Additionally, qNMR is frequently used in quality control to assess the purity and stability of pharmaceuticals without requiring derivatization or destructive processing [7,25]. The European Pharmacopoeia (Ph. Eur.) acknowledges qNMR as a method for purity determination, particularly for reference standard characterization and impurity profiling, while the United States Pharmacopoeia (USP) General Chapter <761> provides guidelines on NMR spectroscopy, but does not yet include extensive qNMR-specific recommendations. Nevertheless, qNMR has been increasingly implemented in pharmaceutical quality control, including in some recent monographs for reference material characterization [32,33]. By providing absolute quantification and structural information in a single analysis, qNMR offers a significant advantage over traditional chromatographic techniques. Interestingly, qNMR contributes to microbiome research by quantifying metabolites such as short-chain fatty acids in fecal samples, shedding light on host–microbiome interactions and their implications for health and disease [34,35]. Its utility extends to nutrition science, where qNMR has been applied to analyze dietary impacts on metabolic profiles, and to environmental toxicology, where it has been used to monitor xenobiotics and their effects on exposed organisms [6,8]. These diverse applications underline the versatility and significance of qNMR as a tool that bridges fundamental biochemical research and translational clinical studies, enabling researchers to unravel complex biological systems with accuracy.

## 4. Practical Aspects

**Figure 2:**
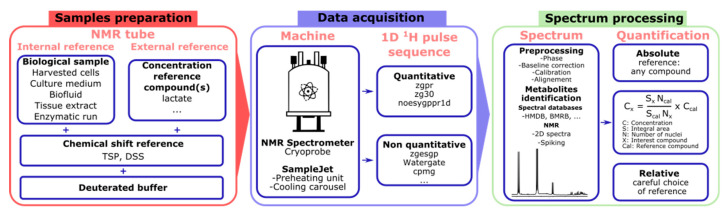
Workflow of a biomolecular NMR study focused on quantifying small molecules. This figure emphasizes the key steps involved, including sample preparation, data acquisition, and spectrum processing.

This section highlights several technical parameters that are critical for a successful quantification study based on NMR measurements. Figure 2 illustrates the typical workflow of a biomolecular NMR study aimed at quantifying small molecules, highlighting the critical steps of sample preparation, data acquisition, and spectrum processing.

### 4.1. Sample Preparation

The choice of sample type, volume, and concentration directly impacts the quality of NMR spectra and the reliability of quantitative analyses. Biofluids like urine, plasma, and saliva, along with cell culture supernatants and metabolite extracts from cell cultures, tissues, and organs, are commonly used in NMR quantification studies [4]. Sample volumes around 200 μL (3 mm tubes) or 550 μL (5 mm tubes) are standard (the volume limit is determined by the active volume of the coil inside the NMR probe and is a characteristic of the probe used), with even smaller volumes achievable using specialized probes [4].

Prompt enzymatic quenching and extraction minimize metabolic evolution and sample degradation, ensuring reproducibility between samples. Several metabolite extraction protocols are available, including methanol extraction or a mixture of methanol, water, and chloroform, followed by drying and storage procedures [36,37,38]. Maintaining proper sample storage conditions, such as storage at −80 °C or in liquid nitrogen, is essential to preserve biological sample integrity and stability [39]. It is important to note that the first freezing process can induce chemical reactions, particularly in the still-liquid phase of partially frozen samples. Such reactions, including those between amines and carbohydrates (e.g., 5-amino salicylic acid with glucose), have been documented and can alter metabolite composition. Therefore, freeze–thaw stability testing is recommended when working with reactive sample matrices to ensure quantification accuracy and sample integrity [40]. The lyophilized sample should be resuspended in buffers just before NMR acquisition, and D_2_O can be used instead of water as a solvent to limit the large and dominating solvent peak in spectra. Pulse sequences with water peak suppression are commonly used (see Section 4.2.2).

pH variations are a significant potential source of quantitative error in NMR analyses. Even small changes in pH (e.g., ± 0.1–0.2 units) can lead to noticeable shifts in chemical shift values, particularly for labile protons, such as those in carboxylic acids or amines. These shifts can impair metabolite identification and quantification, especially when using reference databases or automated software tools like Chenomx, which rely on chemical shift matching. To minimize these errors, samples should be buffered to a well-defined pH—typically 7.0 ± 0.02—for biological studies. NMR samples should thus be buffered to a precise pH and the selection of suitable buffers is crucial for accurate metabolite identification and quantification [37]. Phosphate buffers are commonly used due to their absence of ^1^H signals. If protonated buffers such as Tris or HEPES must be used, deuterated versions are available commercially and can be utilized to avoid introducing undesired peaks. Proper buffering of samples is essential to maintain spectral resolution and chemical stability during acquisition. However, inexperienced users often adjust the pH multiple times, inadvertently increasing salt concentrations, which can lead to changes in pulse width and inaccuracies in qNMR measurements across larger studies. This issue is particularly problematic for cold probes, which are highly sensitive to salt conditions and may exhibit degraded performance under high ionic strength. Additionally, the type of salts used in buffers can influence probe behavior, as conductivity variations can impact sensitivity and heating effects. To minimize these issues, low-conductivity buffers should be considered where possible [41,42].

NMR experiments require the addition of two types of references: chemical shift references, which ensure accurate spectral calibration, and concentration references, which enable quantitative analysis. Chemical shift references, such as TSP (sodium-3-trimethylsilylpropionate-d4) or DSS (sodium-2,2-dimethyl-2-silapentane-5-sulfonate-d6), are commonly used for spectrum alignment, but are not necessarily ideal for absolute quantification due to potential interactions with macromolecules [4].

Concentration references can be implemented using internal or external approaches. Internal referencing involves adding a known-concentration standard directly into the sample before acquisition, whereas external referencing is performed using a separate sample containing a reference compound. Traditional external referencing methods utilize coaxial inserts, where the reference compound is placed in a separate compartment within the same NMR tube, preventing direct interactions with the sample while still providing a calibration standard [22]. While the coaxial capillary method provides a reliable external reference, its use can be challenging due to delicate handling requirements.

To address these practical constraints, alternative external referencing approaches such as PULCON (Pulse Length-based Concentration Determination) and ERETIC (Electronic Reference To access In vivo Concentrations) have been developed.

These methods use separately acquired calibration spectra to establish a concentration reference, which can then be applied to analyze the study sample. This allows for accurate quantification without physically introducing a reference compound into the sample or using an insert. PULCON can be used with any 1D pulse sequence, provided that the reference sample and the study sample are acquired under identical conditions, including the same pulse sequence and acquisition parameters. The fundamental principle behind PULCON is the accurate determination of concentration ratios by comparing signal intensities across matched experimental conditions, making it a highly versatile approach in qNMR applications. Similarly, ERETIC 2 is a more flexible referencing method that offers an alternative way to correct for instrumental and acquisition variabilities [25,43].

Table 1 provides an overview of the key experimental protocols and highlights the critical points to consider. In addition to concentration limits, it is important to consider the dynamic range of the sample, particularly in metabolomics workflows where components may span several orders of magnitude in abundance.

### 4.2. NMR Data Acquisition

#### 4.2.1. Acquisition Temperature

As most biological samples are sensitive to temperature, samples are typically stored at 4 °C before acquisition. The use of a Bruker SampleJet system (Bruker Biospin AG, Fällanden, Switzerland), which allows for keeping the samples refrigerated before analysis, is preferred as it also allows for automated sample changes, eliminating the need for manual intervention, especially during overnight experiments. To enhance signal sensitivity, it is beneficial to conduct the acquisition at higher temperatures. The standard range of temperature for NMR spectra acquisition is well-documented, most often between 303 K and 310 K, striking a balance between increased signal sensitivity and maintaining sample and molecule integrity [4].

#### 4.2.2. Which Pulse Sequence?

To obtain quantitativeness, pulse sequences must be chosen carefully, as excitation pulses (typically around 10 µs) must be short and uniform across the entire spectral width of interest [23]. As biological samples are commonly dissolved in aqueous solutions, the pulse sequences must integrate special water suppression techniques, such as water presaturation, to suppress the peak from water that would otherwise dominate spectra [24,50]. In contrast, when samples are prepared in non-aqueous solvents, such as CDCl_3_ or DMSO-d_6_ (e.g., for lipid extracts or hydrophobic compounds), water suppression is not necessary, and simpler pulse sequences—such as zg or zg30—may be more appropriate. These sequences avoid unnecessary presaturation steps and can improve excitation homogeneity and acquisition speed under non-H_2_O conditions. Additionally, when using presaturation in H_2_O-based solvents, it is important to recognize that signal attenuation is not limited to resonances near the water peak. Labile protons, such as those found in –OH, –NH, or –NH_2_ groups, may undergo solvent exchange with water or be affected by cross-relaxation with the saturated water magnetization. As a result, their signal intensities can be significantly reduced or even completely suppressed, even if they are not directly overlapping with the presaturation frequency. Since such exchangeable protons are commonly encountered in biological samples, they are generally unsuitable for quantitative integration in presaturation-based experiments. The selection of the pulse sequence should thus be adapted to the solvent system used, balancing the need for water suppression with the goal of maximizing quantitativeness. Moreover, it is important to note that, to achieve accurate quantification, users must maintain consistent NMR experiments (pulse sequence and parameters) throughout the study to eliminate the impact of phase cycling, which varies between sequences and induces changes in NMR signal intensity.

Table 2 provides an overview of popular pulse sequences for the study of biological small molecules and metabolites, emphasizing their potential to deliver quantitative results when appropriate acquisition parameters are set. While PFG elements are present in many sequences commonly used in metabolomics, their presence alone does not preclude quantitativeness. For example, the noesygppr1d sequence includes PFGs for solvent suppression and phase cycling, but when used with appropriate parameters (e.g., full relaxation delay, uniform excitation), it can yield accurate quantitative results. In contrast, other PFG-based sequences—such as diffusion-edited or excitation-sculpting sequences—often involve filtering or relaxation-weighted components that make them unsuitable for quantification. This distinction is clarified in Table 2. Even though Bruker sequences are presented in Table 2, it is important to note that equivalent sequences from other manufacturers, such as Jeol or Agilent, may offer similar capabilities. One of the most employed pulse sequences that fulfils the quantitative criteria is the [delay–pulse–acquisition] sequence (Bruker zgpr sequence). In metabolomics, two pulse sequences that integrate more advanced strategies for improved signal detection and water suppression are commonly employed: nuclear Overhauser effect spectroscopy (NOESY) and Carr–Purcell–Meiboom–Gill (CPMG) [4,51].

NOESY with presaturation selective water suppression is considered quantitative as long as the recycle delay between acquisitions is long enough (in practice, 30–60 s) [52]. The 1D NOESY presaturation sequence (noesygppr1d) is the recommended pulse sequence for use with Chenomx software, which provides a curated library of reference compound spectra acquired under standardized conditions. To ensure compatibility with this database and achieve reliable metabolite quantification, NMR spectra should be acquired at a temperature of 298 K (25 °C) using a 90° excitation pulse, with an acquisition time of 4 s and recycling delay d_1_ including a 990 ms saturation pulse on water. A spectral width of 12 ppm, along with 32–128 scans depending on sample concentration, is advised. The use of TSP or DSS as an internal chemical shift reference (typically at 0.5–1 mM) and consistent pH adjustment to 7.00 ± 0.02 are essential, as the software relies on precise chemical shift matching. Strict adherence to these parameters enables accurate spectral deconvolution and quantification within Chenomx’s interface.

In the case of complex mixtures, such as plasma samples, for example, the presence of macromolecules such as proteins or large amounts of lipids can manifest as broad signals that may hide signals from metabolites of interest or significantly distort the baseline.

The CPMG spin–echo pulse sequence leverages the significant differences in transverse (T_2_) relaxation times between small molecules and macromolecules, effectively suppressing NMR signals from proteins and other large biomolecules. The resulting 1D ^1^H-NMR spectrum closely resembles a NOESY spectrum obtained after macromolecules are removed via methanol extraction. However, caution is required when using the CPMG sequence, as any metabolites bound to proteins or other macromolecules may experience signal loss, leading to inaccurate quantification. Also, as longer pulses and relaxation delays are used, inducing a non-uniform relaxation across the signal, the CPMG is not considered a quantitative sequence [4].

#### 4.2.3. Signal-to-Noise Ratio, Detection Limits, and Accuracy

The limit of quantification (LOQ) in quantitative NMR is fundamentally determined by the signal-to-noise ratio (SNR), rather than by an absolute concentration threshold. Typically, a minimum SNR of 10:1 is required for quantification, though a ratio of 3:1 may be acceptable for detection (LOD). Achievable LODs depend on the spectrometer field strength, probe type, and sample matrix. For example, with a 600 MHz cryoprobe, LODs can reach approximately 5 µM for amino acids, 20 µM for carbohydrates, and 50 µM for lipids in aqueous biofluids [53]. These values may vary depending on relaxation properties and matrix complexity. Guidelines for assessing and validating LOQ and LOD values have been proposed, including those presented in the *qNMR Guideline Version 001* [54], which provides a practical framework for evaluating detection thresholds, quantification precision, and accuracy.

**Figure 3:**
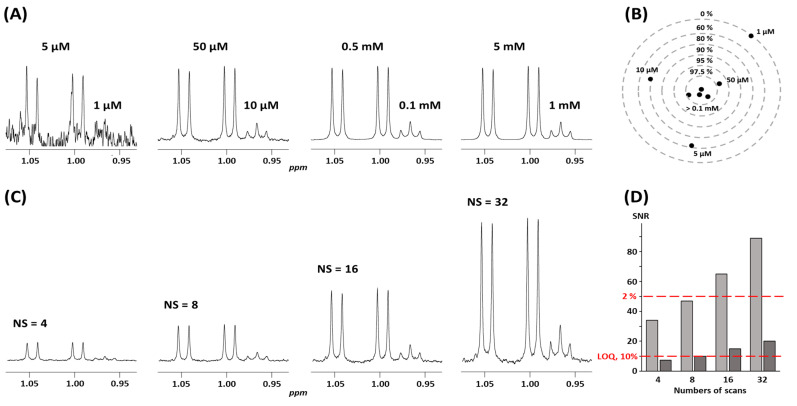
Quantitative NMR measurements. Spectra were recorded in D_2_O at 600 MHz on a Bruker Avance NEO spectrometer equipped with a 5 mm HPCN QCI cryoprobe. (**A**) ^1^H NMR spectra (16 scans) focused on methyl signals for increasing concentrations of valine (5 µM, 50 µM, 0.5 mM, 5 mM) and leucine (1 µM, 10 µM, 0.1 mM, 1 mM). Spectra are scaled according to the maximum intensity of the signals for valine. (**B**) Schematic representation on a logarithmic scale of the trueness of the quantification, comparing the measured values and the true value for all concentrations studied. (**C**) Spectra showing the variation in the number of scans (4, 8, 16, 32) for a solution containing valine (50 µM) and leucine (10 µM). (**D**) Bar chart representing signal-to-noise ratio (SNR) as a function of the number of scans. Red lines indicate the required SNR for the limit of quantification (LOQ) with a standard deviation (SD) of 10% and 2%.

To ensure measurements with acceptable accuracy, i.e., precision and trueness, an NMR spectrum needs to achieve a sufficient signal-to-noise ratio (SNR) [15]. The SNR is influenced by various experimental factors, including the magnetic field strength (B0) of the NMR spectrometer, the homogeneity of the field and quality of the shims, the sample concentration, the number of scans (NS), and the probe temperature [4,24]. Opting for a higher magnetic field strength is generally advantageous, as it enhances both sensitivity and spectral resolution [30]. NMR spectrometers up to 800 MHz are widely available and sometimes equipped with high-throughput accessories such as the Samplejet apparatus that allows for cold storage of the sample and automated acquisitions. In the field of metabolomics, a 600 MHz spectrometer is often considered a “sweet spot” compromise between resolution and accessibility, while a 400 MHz spectrometer can suffice for targeted analysis of non-complex mixtures. To further enhance sensitivity, cryoprobes reduce electronic thermal noise by cooling the probe and its electronics to near liquid helium or nitrogen temperatures (Prodigy probes). Cryoprobes can reach up to a four-fold improvement in SNR, leading to higher-quality spectra, faster data acquisition, and the detection of lower-concentration samples [4]. The SNR depends on the number of scans NS. As the NMR signal is weak, a way to improve the SNR consists of accumulating and summing multiple scans from the same sample. To visually illustrate this principle, Figure 3 presents the effect of varying concentrations of valine and leucine, as well as the number of scans, on the resulting NMR spectra and the corresponding SNR. The enhancement is proportional to the square root of the number of experiments performed. Achieving a high SNR is thus possible at the trade-off with experiment duration. NS is a central parameter that must be chosen experimentally; it greatly impacts the desired SNR, acquisition time, and cost associated with NMR access. The optimal NS value may vary depending on the spectrometer, the concentration of the molecules to quantify, and the level of accuracy, but numbers between 16 and 256 (or even higher) scans are very common, for a total duration of NMR acquisition ranging from 2 to 45 min for each sample. The impact of the molecule concentrations on the accuracy of the quantification results is shown in Figure 3B, which demonstrates that low concentrations (micromolar range) are more challenging to determine and prone to errors than higher concentrations. The center of the target, corresponding to higher concentrations (millimolar range), shows a high level of accuracy. As the concentration decreases, points move outward on the target, and the accuracy diminishes, showing lower trueness at lower concentrations (micromolar range). This visualization highlights the challenges associated with accurately quantifying low concentrations, emphasizing the increased error as concentrations decrease.

Quantification accuracy depends on the SNR of an experiment, and thus on its total duration. Figure 3 highlights the relationship between the number of scans and the accuracy of quantification. Higher SNR leads to more precise and reproducible results (Figure 3D). In practice, accurate and reliable quantification is achieved when concentrations reach a few tens of micromolars [4]. Quantifying lower-concentration metabolites requires significantly longer data acquisition times that are often unrealistic for large sample cohorts (refer to the Section 4. These trade-offs between sensitivity, acquisition time, and experimental feasibility are at the core of determining meaningful LOQ thresholds in qNMR workflows.

#### 4.2.4. Experiment Parameters

The total duration of an NMR experiment is influenced by several acquisition parameters, including the number of scans, the relaxation delay between two experiments (d_1_), the excitation pulse sequence, and the acquisition time.

As highlighted previously, to ensure quantitative measurements, the delay between two scans should be sufficient so that the NMR signals of the sample molecules have reached a fully relaxed state. Experimentally, this is obtained by choosing a d_1_ value that is at least five times the T_1_ value. To determine an appropriate repetition delay for a specific sample, it is strongly recommended to measure the T_1_ relaxation time of representative resonances within the spectrum. This can be achieved using standard inversion–recovery or saturation–recovery experiments, which are available in most commercial NMR software libraries (e.g., Bruker’s t1ir, t1rho (Bruker Biospin AG, Fällanden, Switzerland), or equivalent pulse sequences). These measurements are relatively quick to set up and can significantly improve the accuracy of quantitative results by ensuring that full relaxation occurs between scans. Once T_1_ values are known, the recycle delay (d1) can be confidently set to at least 5 × T_1_ for full relaxation or adjusted according to the desired trade-off between accuracy and throughput. An alternative pragmatic approach consists of acquiring and comparing a series of spectra with a range of d_1_ values and checking that peak intensities do not increase with longer d_1_ delays. Commonly, delays of up to 5 to 60 s are necessary between two scans to obtain quantitative acquisition conditions. As most experiments require 16–256 scans to reach an SNR sufficient to evaluate low-concentration species, increasing the d_1_ delay between two scans significantly increases the total acquisition time for each spectrum and compromises the throughput for sample cohorts. The delay between two scans can be reduced by employing a flip angle of 30°, rather than 90°, for the excitation pulse, which reduces the d_1_ delay to rebuild the signal, but also reduces the intensity of the detected signal, but at the cost of signal loss. Alternative strategies consist of adding a relaxation agent in the sample to accelerate relaxation [55]. Unfortunately, compromises on d_1_ duration are frequently made to maintain acceptable acquisition durations and throughput, leading to the non-quantitative acquisition conditions discussed in this article. Such trade-offs are often used in metabolomics and require adapted quantification strategies.

### 4.3. Data Exploitation

Quantification in NMR is based on signal integration and comparing the integrated areas to that of a concentration reference. If the conditions for absolute quantification are met, direct quantification can be achieved by comparing the peak areas of a compound of interest to a single concentration standard. If these conditions are not met, the use of one molecule to determine the concentration of another molecule is not possible. However, comparing integrals from the same compound between a series of samples is always possible and the percentage of variation for a single molecule can be obtained, provided the spectra were acquired under the same conditions. Additionally, the use of a concentration reference for that particular molecule can be used to determine the concentration. The conditions necessary to achieve either absolute or relative quantification in NMR are summarized in Table 3, which outlines the key parameters to consider for accurate measurements.

Accurate quantification also relies heavily on the careful pre- and post-processing of NMR spectra. Baseline correction is typically performed using polynomial fitting of low order (usually first to third), which removes slow baseline drift without distorting peak areas. Cautious phasing, preferably carried out manually, is essential to maintain symmetrical peak shapes and avoid the introduction of negative lobes that can compromise integration. Signal integration itself must be applied consistently, using well-defined chemical shift windows that cover the majority of the peak area (usually 80–90% of the peak height), ensuring reliable comparison across samples. In regions where peak overlap occurs and in the case of complex mixtures, peak overlaps can hinder the application of direct integral comparison methods. To address this challenge, several peak deconvolution strategies have been proposed to analyze the Lorentzian line shapes [56] and identify individual peak contributions to the overlapping multiplets. Computational methods exist to assist in the identification and quantification of compounds in biological samples [57]. Examples of such methods include Chenomx [25], AMIX [57], Batman [13], and online tools, like NMRProcFlow [58]. Chenomx has been accepted as a gold-standard solution in metabolomics for concentration estimation under non-quantitative, but high-throughput conditions. ChenomX uses a library of pure-compound spectra acquired under very precise acquisition conditions, avoiding the need to use multiple concentration standards (i.e., one for each molecule to be studied). Provided that experimental spectra are acquired using strictly the recommended acquisition parameters, ChenomX can be used to deconvolute, assign, and determine the concentrations of molecules with a complex sample, even if peak overlays occur. The accuracy of quantitative NMR methods depends heavily on both the acquisition parameters and the strategy used for signal analysis. Traditional qNMR based on direct integration under fully quantitative conditions (e.g., 90° pulse, ≥5 × T_1_ relaxation delay, internal or external reference) can yield excellent results, with reported trueness >98.5% and precision typically within 5% [9]. However, this approach requires non-overlapping peaks and well-controlled acquisition, making it less feasible for complex biological matrices. In such cases, spectral deconvolution is often necessary. Software like Chenomx uses a database of reference compound spectra acquired under defined conditions and fits them to experimental spectra via line shape modeling. While less accurate than absolute qNMR, Chenomx can provide reproducible concentration estimates in highly complex matrices with reported errors often <10–15% when acquisition is performed according to protocol [4]. Deconvolution approaches such as BATMAN (Bayesian AuTomated Metabolite ANalyzer) model overlapping peaks probabilistically and are particularly useful in high-throughput untargeted metabolomics, though they may require more parameter tuning and computational resources. Their accuracy depends on signal complexity and prior information, and typical relative quantification errors can range from 10% to 25%. While Chenomx is more suited to targeted profiling under standardized conditions, tools like BATMAN or NMRProcFlow offer flexible alternatives when working with broader, less-defined mixtures. Ultimately, the choice of method involves a trade-off between quantification accuracy, spectral complexity, and practical throughput constraints.

## 5. Challenges and Limitations of Quantitative NMR

Despite its numerous advantages and recent developments, qNMR faces several technical and practical challenges that can hinder its widespread adoption, particularly among non-specialists. One significant limitation is the inherent low sensitivity of NMR. This limitation restricts the detection of low-concentration metabolites, requiring prolonged acquisition times and high-field spectrometers equipped with cryogenic probes to enhance sensitivity. Additionally, signal overlap in complex biological mixtures, such as serum or urine, complicates the accurate quantification of individual metabolites. To address these issues, advanced computational tools like Chenomx, Bayesil, and BATMAN have been developed to deconvolute overlapping signals and extract quantitative information with greater accuracy [12,13,25]. This challenge is further exacerbated by the presence of broad macromolecular signals, which can obscure small-molecule peaks. Furthermore, the binding of commonly used reference compounds like DSS or TSP to proteins in biological samples can distort peaks, complicating peak integration or deconvolution and reducing quantification accuracy. Another critical challenge lies in the standardization of sample preparation and acquisition protocols. While NMR measurements are recognized as very reproductible, variability in sample handling, buffer conditions, and instrument settings can lead to discrepancies in results across laboratories. Standardized workflows and calibration-free methods, such as PULCON and ERETIC2, have been proposed to mitigate these issues and improve reproducibility [25,43]. Furthermore, efforts to harmonize experimental conditions, including the adoption of universal acquisition parameters and guidelines, aim to enhance inter-laboratory consistency.

One of the primary challenges encountered when dealing with complex mixtures is the issue of signal overlap, which can hinder the identification of biomolecules. To address this, 2D NMR spectroscopy proves to be a valuable solution as it enhances signal dispersion and resolution. Several commonly used 2D pulse sequences include the HSQC (heteronuclear single quantum coherence) for detecting one-bond ^13^C-^1^H spin–spin couplings, correlation spectroscopy (COSY), or total correlation spectroscopy (TOCSY) for identifying correlations between protons, and two-dimensional J-resolved (J-RES) spectroscopy [59]. Quantification using 2D methods presents additional challenges, and while some sequences have been developed [60,61,62], their practical application to complex biological mixtures remains challenging [63]. Ongoing advancements in NMR instrumentation and methodology are also addressing these limitations. The development of ultra-high-field spectrometers and hyperpolarization techniques, such as dissolution dynamic nuclear polarization (DNP), offers significant sensitivity enhancements, enabling the detection of metabolites at nanomolar concentrations [9,64]. Additionally, the integration of NMR-visible nuclei, such as ^15^N, ^31^P, or ^19^F, can enable the specific tracking of a drug’s fate within an organism or tissue, along with multidimensional spectra [4,30]. In metabolomics, ^1^H–^13^C decoupled spectra are commonly employed to aid in the identification and quantification of metabolites due to their larger spectral width, which reduces signal superposition. ^15^N is used to investigate nitrogen-containing functional groups in metabolites, while ^31^P is particularly useful for analyzing phosphorylated compounds, including lipids [65]. However, quantifying and monitoring these nuclei in their natural abundance can be challenging. Isotope labeling presents a viable solution for isotopic tracking or tracing experiments, aiding in the elucidation of synthetic pathways, such as glycolysis or the TCA cycle [66,67]. Nonetheless, when it comes to metabolite quantification, ^1^H acquisition spectra remain the preferred method due to their efficiency and relatively straightforward implementation.

Moreover, the integration of artificial intelligence and machine learning algorithms holds promise for automating spectral analysis and peak assignment, thereby simplifying data interpretation and reducing reliance on expert users [68]. By overcoming these challenges, qNMR can realize its full potential as a robust and accessible tool for quantitative metabolomics and biomedical research.

## 6. Conclusions

By prioritizing practical guidance and accessibility for non-specialists, this review distinguishes itself from prior work that emphasizes advanced methodologies or niche applications. This approach aims to foster interdisciplinary collaboration, making quantitative NMR more accessible to a broader audience and facilitating its adoption in biomedical and biological research. By focusing on targeted metabolite quantification or studying their variations, for example, ^1^H NMR analysis can provide valuable insights into biological and medical questions. This article provides essential experimental recommendations for non-specialists, including careful sample preparation, selection of appropriate solvents (buffers), choice of reference, and optimization of acquisition parameters (sequences, delays). These practical guidelines aim to help researchers to achieve more reliable and reproducible results. Overall, quantitative NMR offers a robust and accessible tool for biologists to enhance their research capabilities. Its versatility in analyzing a wide range of metabolites and xenobiotics, coupled with its non-destructive nature, makes it an indispensable method for advancing our understanding of complex biological systems. By integrating NMR techniques into their research, biologists can achieve precise, reliable, and comprehensive data that supports more informed decision-making and scientific discovery. The continued development and application of quantitative NMR will undoubtedly contribute to significant advancements in fields such as pharmacology, toxicology, and metabolic research, fostering innovation and progress in understanding the intricate biochemical networks that underlie health and disease.

## Figures and Tables

**Table 1:** Overview of biological material, quantity, and preparation compatible with quantitative NMR in the case of routine measurements at 400–800 MHz.

Parameter	Specification	Comments
Biological sample	Biofluids: urine, plasma, saliva, cells supernatant	For mice, urine and serum volumes might reach more than 50 µL, while for humans, volumes might be higher. Thus, dilution in buffers, preferentially deuterated, is often needed to complete the total volume needed in an NMR tube [44].
	Cells lysate	The resuspension of metabolites extracted from 2 to 10 million cells typically yields a satisfactory signal-to-noise ratio [38].
	Tissues	20 to 150 mg of tissues are commonly used for NMR acquisition. Metabolites are extracted and resuspended in deuterated buffers [45]. When quantifying metabolites in tissue samples, additional considerations are necessary to ensure accuracy and reproducibility. Tissue weighing precision, water content variability, and metabolite extraction efficiencies can introduce variability in concentration measurements and should be carefully controlled [18,36]. Additionally, repeatability assessments and extraction validation should be performed to ensure quantitative reliability across different samples and biological replicates. Comprehensive discussions on optimized tissue preparation protocols for qNMR can be found in previously established guidelines [37,39].
Total volume	200 µL or 550 µL	The sample size depends on the characteristics of the NMR probe used. Most often, 5 mm probes can accommodate 3 mm tubes with appropriate spinners or adapters. To minimize the solvent contribution to spectra, more concentrated biological material in a 3 mm tube is preferred, rather than diluting samples to fit into 5 mm tubes. NMR is non-destructive: samples can be used for other purposes after NMR analysis.
Small-molecule concentration range	10 µM to 50 mM	Higher concentrations allow for faster acquisition and better signal-to-noise ratios, but mixtures often contain components spanning several orders of magnitude in concentration. In metabolomics, for example, certain metabolites may be present at sub-µM levels, while others exceed 10 mM. This large dynamic range poses a challenge to quantification, as high-concentration signals may obscure weaker ones and saturate the receiver. Pulse sequences and processing strategies should be adapted accordingly to preserve quantitative accuracy across the entire concentration range. Pre-fractionation or targeted profiling can help to address this issue in complex samples.
Protein content	As low as possible	A lower protein content is preferable. Acquisition schemes, such as those inserted into the CPMG pulse sequence, can reduce the impact of the protein broad spectral background at the cost of lowered sensitivity and accuracy. Samples can be filtered using low-molecular-weight cutoffs to remove proteins. Low protein concentrations and catalytic amounts are usually not an issue [46].
Additives	Flexible, with the same 10 µM to 50 mM concentration limit	Caution should be taken with protonated molecules that may have peaks overlaying important signals, such as buffers and common additives, or contaminants, such as DMSO, glycerol, methanol, etc.
Chemical shift reference	10 µM to 1 mM	TSP or DSS are the most common. Some metabolites from the sample, such as glucose or alanine, can also be used as references [47].
Concentration reference	See below	High-quality concentration standards should be used for accurate quantification. Concentration references can be internal (added directly to the sample or placed inside a capillary immersed in the sample) or external (in another NMR tube). Lactate solutions are often used as they are available commercially at standardized concentrations. TSP or DSS can be used as concentration references, but they tend to bind proteins in biological samples, such as serum or urine, which alters their linewidth. In this situation, a strategy based on diluting untreated samples in deuterated solvents can be used to precipitate proteins and recover metabolites quantitated relative to standard reference compounds, such as DSS [48]. Alternative standards, such as Certified Reference Materials [49], are widely used in the pharmaceutical field to ensure compliance with regulatory guidelines [49].
Sample quality	Homogeneous	Samples can be centrifuged or filtered if necessary. Be cautious of glycerol contamination from 0.5 µm filters, as well as acetate or formate originating from various lab consumables.
Solvent	Deuterated solvents (e.g., D_2_O, CDCl_3_)	The final NMR solvent must contain at least 5 to 10% deuterium for the spectrometer to compensate the magnetic field drift over the time of the NMR experiment. Typically, 5–10% D_2_O is added to aqueous samples. Cellular and tissue extracts often separate into polar and non-polar fractions. Dried samples from polar fractions can be resuspended in 100% D_2_O buffered solutions. Hydrophobic phases resulting from metabolite extractions can be dissolved in organic solvents such as CDCl_3_.
pH and buffers	Adjust to the required pH	As pH influences chemical shifts, adequate buffers should be used to minimize pH variations between samples; proton-less buffers such as phosphate are most commonly used in the range from 20 to 200 mM. Lower concentrations of salts are preferred, as low-conductivity buffers favor higher NMR sensitivity [41].
Sample storage	Store under appropriate conditions	Store biological samples at low temperatures (−80 °C or in liquid nitrogen) to prevent metabolite evolution or degradation. Avoid repeated freeze–thaw cycles. NMR samples can be kept at a low temperature (4 °C) before acquisition.
Acquisition temperature	4 °C to 90 °C	Higher temperatures allow for better NMR signal sensitivity. Temperatures of 30–37 °C are most common in metabolomics and enzymology. Low temperatures should only be used in proven cases of instability.

**Table 2:** Overview of popular pulse sequences for the study of biological small molecules and metabolites, highlighting their potential to deliver quantitative results (if proper acquisition parameters are set) and their main features. For non-aqueous solvents, presaturation is not required and simpler sequences (e.g., zg, zg30) are preferred. These offer better excitation profiles and reduce unnecessary delays in the absence of water signal.

Bruker Pulse Sequence or Tools	Considered Quantitative Provided That a Sufficiently Long Recycle Delay Is Used	Description, Applications
Zgpr	Yes	The standard 1D proton NMR pulse sequence with water presaturation is commonly used for the routine analysis of small molecules in metabolomics. However, signals close to the presaturation frequency may experience a loss of intensity.
Zgpr30	Yes	A variant of zgpr with a 30° flip angle that allows for reducing the relaxation delay (d_1_). Useful when faster data acquisition is needed, but at the cost of a lessened sensitivity. PULCON method cannot be used with this sequence.
Noesygppr1d	Yes	NOESY sequence with presaturation to suppress water. Routine spectrum in metabolomics.
Cpmgpr1d	No	Includes a CPMG sequence to suppress broad signals from macromolecules. Suitable for samples that display altered baseline due to the background contribution of lipids or proteins.
zgesgp	No	1D ^1^H excitation sculpting (ES) with gradients. Uses shaped pulses and gradients to selectively excite and suppress specific signals. Effective water suppression and improved detection of overlapping signals.
Diffusion-edited NMR (ledbp2s1d)	No	Suppresses signals from small molecules by exploiting differences in diffusion rates. Useful for complex mixtures and identifying macromolecules.
Most 1D NMR sequences with pulsed field gradients (PFG)	No (except in some cases)	PFG techniques are used to improve solvent suppression and reduce artifacts. However, in many applications (e.g., diffusion-editing or selective excitation), PFG elements can interfere with signal intensity or introduce relaxation-based biases, precluding quantitative use. Notably, some PFG-containing sequences, such as noesygppr1d, can be quantitative under appropriate acquisition conditions.

**Table 3:** Conditions for NMR acquisition, including common pulse sequences, repetition times, and quantitative conditions. This table distinguishes between quantitative and non-quantitative approaches, outlining the necessary parameters for accurate quantification of small molecules, such as the use of reference compounds and specific acquisition times. It also highlights the conditions under which quantification can occur in the absence of standards, as well as the recommendations for utilizing Chenomx software (Chenomx, Edmonton, AB, Canada).

Conditions of NMR Acquisition	Common Pulse Sequence	Repetition Time	Chemical reference	Exploitation of Data, Quantification and Comments
Quantitative conditions	Short (zgpr, zgpr30, noesygppr1d)	At least 5 × T_1_ (e.g., 5–60 s depending on compound). T_1_ should be measured using inversion–recovery or saturation–recovery methods.	A single-concentration reference compound (internal, spiked, or external) can be used to determine the concentration of any other compound	Absolute quantification by integration and Equation (1). For pulses of less than 90°. the repetition time can be shortened (e.g., 3 T_1_ for a 30° pulse with the zgpr30 pulse sequence).
Non quantitative conditions	Any sequence, short or longer (cpmgpr1D)	Below 5 T_1_: 2–4 s between experiments	One standard for each quantified compound (spiking experiment or external reference)	Relative quantification by integration and absolute quantification by direct comparison with the same molecule at a known standard Possible in the absence of standard: determination of variation by comparison of same peak integrals between a set of spectra.
Chenomx	noesygppr1D, cpmgpr1d	4 s	One compound (TSP at known concentration)	Strict adherence to Chenomx acquisition parameters recommendations. Dedicated software and peak-fitting tool. Limited library of biomolecules.

## Data Availability

Data sharing is not applicable.
